# Scrub Typhus Meningitis in South India — A Retrospective Study

**DOI:** 10.1371/journal.pone.0066595

**Published:** 2013-06-14

**Authors:** Stalin Viswanathan, Vivekanandan Muthu, Nayyar Iqbal, Bhavith Remalayam, Tarun George

**Affiliations:** 1 Department of General Medicine, Indira Gandhi Medical College, Kathirkamam, Pondicherry, India; 2 Department of Internal Medicine, Pondicherry Institute of Medical Sciences, Kalapet, Pondicherry, India; Charité University Medicine Berlin, Germany

## Abstract

**Background:**

Scrub typhus is prevalent in India although definite statistics are not available. There has been only one study on scrub typhus meningitis 20 years ago. Most reports of meningitis/meningoencephalitis in scrub typhus are case reports

**Methods:**

A retrospective study done in Pondicherry to extract cases of scrub typhus admitted to hospital between February 2011 and January 2012. Diagnosis was by a combination of any one of the following in a patient with an acute febrile illness- a positive scrub IgM ELISA, Weil-Felix test, and an eschar. Lumbar puncture was performed in patients with headache, nuchal rigidity, altered sensorium or cranial nerve deficits.

**Results:**

Sixty five cases of scrub typhus were found, and 17 (17/65) had meningitis. There were 33 males and 32 females. Thirteen had an eschar. Median cerebrospinal fluid (CSF) cell count, lymphocyte percentage, CSF protein, CSF glucose/blood glucose, CSF ADA were 54 cells/µL, 98%, 88 mg/dL, 0.622 and 3.5 U/mL respectively. Computed tomography was normal in patients with altered sensorium and cranial nerve deficits. Patients with meningitis had lesser respiratory symptoms and signs and higher urea levels. All patients had received doxycycline except one who additionally received chloramphenicol.

**Conclusion:**

Meningitis in scrub typhus is mild with quick and complete recovery. Clinical features and CSF findings can mimic tuberculous meningitis, except for ADA levels. In the Indian context where both scrub typhus and tuberculosis are endemic, ADA and scrub IgM may be helpful in identifying patients with scrub meningitis and in avoiding prolonged empirical antituberculous therapy in cases of lymphocytic meningitis.

## Introduction

Scrub typhus is an acute febrile illness caused by *Orientia tsutsugamushi*, and is characterized by an eschar, lymphadenopathy, multisystem involvement and a rapid response to doxycycline. Scrub typhus is seen in all terrains of the tsutsugamushi triangle, a geographical region of south and east Asia and the southwest Pacific and is related mostly to agricultural activities [1]. Although it was known in China in the 3^rd^ century A.D, scrub typhus (tsutsugamushi fever) was described by Hakuju Hashimoto in 1810 in people living on the banks of the Shinano river [2] and later by Baelz and Kawakami in 1879 [3] as Japanese “flood fever” [4]. Tsutsugamushi describes a Japanese term for *tsutsuga* “illness” and *mushi* “insect/creature”. Taiwan is the centre of the tsutsugamushi triangle and the first case reported in that country was in 1915 [5]. The first and second cases in Korea were reported in 1951 and 1986 respectively [6] and it now has the highest reported incidence in the world [7]. About one million new cases are identified annually [8]. The first reported cases in India were in 1934, in Himachal Pradesh [4]. We do not have definite statistics in India due to lack of awareness, unavailability and high cost of diagnostic kits and the fact that it is not a reportable illness.

The larval forms (chiggers) of the trombiculid mite transmit the disease to humans and other vertebrates [9]. The mite has four life cycle stages: egg, larva, nymph and adult [9]. Horizontal transmission occurs in rodents and humans get accidentally infected following bites of chiggers [10]. Vertical transovarial transmission occurs in mites [10] although one case of transplacental spread has been reported in a pregnant woman who delivered a preterm baby with hepatosplenomegaly, meningitis, sepsis and scrub IgM positivity [11]. *Leptotrombidium deliense* and *L. scutellare* are the mites for the summer type (March to November) and winter type (September to December) scrub typhus respectively. Correspondingly, the reservoir hosts (rodents) include *Rattus losea, R. flavipectus*, and *Apodemus agrarius* for the former and *A. agrarius, Cricetulus triton*, and *R. Norvegicus* for the latter [12]. There are more than 30 antigenically different strains apart from the 6 important serotypes of *O. tsutsugamushi* – Gilliam, Karp, Kato, Shimokoshi, Kawasaki, and Kuroki [13]. Infection spreads through both hematogenous and lymphatic routes [12]. Target site for multiplication are the endothelial cells of the various systems [14]. Both humoral and cell mediated immunity are important for combatting scrub typhus [14].

Chills and fever occur by the 3–4^th^ day of bite, and rash and lymphadenopathy appear at end of the first week [13]. Incubation period ranges from 6–20 days [15]. Serious complications occur during the second week of illness and comprise of pneumonitis, pleural effusion, hepatomegaly, edema, acute kidney injury(AKI), acute respiratory distress syndrome(ARDS) and meningitis [16]. Most studies of meningitis and meningoencephalitis in scrub typhus are case reports/series (Table 1). Silpapojakul *et al.*, in 1991 described nine patients(9/72) presenting with meningitis in the earliest study of rickettsial meningitis [17]. CSF studies were similar to tuberculous meningitis(TBM) and viral etiologies [17]. Although literature describing various neurological complications in scrub typhus was available (Table 1), we could not find any previous studies on scrub typhus, from India or elsewhere that compared patients with and without meningitis.

**Table 1:** Neurological complications in scrub typhus.

	No	Year	Authors	Reference	Country	Diagnosis
1	1	1988	Lin et al	Zhonghua Min Guo Xiao Er Ke Yi Xue Hui Za Zhi	China	Meningitis
2	1	1991	Kuroda et al	Nihon Naika Gakkai Zasshi	Japan	Meningitis
3	9	1991	Silpapojakul et als	Arch Int med	Thailand	Meningitis; meningoencephalitis
4	2	1991	Silpapojakul et al	Pediatr Infect Dis J	Thailand	Meningitis
5	1	1992	Ting et al	J Formos Med Assoc	Taiwan	Brachial plexus neuropathy
6	25	1997	Pai et al	Clin Infect Dis	Korea	Meningitis
7	1	1997	Fang et al	J Formos Med Assoc	Taiwan	Meningitis; seizures
8	1	1999	Ben et al	J Microbiol Immunol Infect	Taiwan	Meningoencephalitis
9	1	1999	Chua et al	Neurol J Southeast Asia	Malaysia	Confusion; periventricular hyperintensities
10	2	2000	Kim et al	J Korean Neurol Assoc	Korea	Meningitis
11	1	2000	Kim et al	Arch Neurol	Korea	Encephalomyelitis; bilateral abducens ;facial nerve palsy, anarthria, dysphagia; quadriparesis
12	1	2002	Tsay et al	QJM	Taiwan	Meningoencephalitis, shock
13	1	2003	Sirisanthanaet al	Pediatr Infect Dis J	Thailand	Meningitis
14	5	2003	Mathai et al	Ann. N.Y. Acad. Sci	India	Altered sensorium
15	6	2004	Silpapojakul et al	Trans R Soc Trop Med Hyg	Thailand	Encephalitis and meningitis
16	1	2005	Yang et al	Tzu Chi Med J	Taiwan	Intracranial hemorrhage
17	6	2006	Premaratnaet al	Clin Infect Dis	Sri Lanka	Sensorineural hearing loss; tinnitus; encephalitis
18	3	2006	Mahajan et al	Emerg Infect Dis	India	Meningitis
19	16	2006	Mathai et al	J Infection	India	Altered sensorium
20	1	2006	Chen et al	Acta Neurol Taiwan	Taiwan	ADEM
21	1	2007	Wang et al	Am J Trop Med Hyg	Taiwan	Seizures
22	1	2007	Moon et al	Infect Chemother	Korea	Subdural hematoma
23	1	2007	Lee et al	Scand J Infect Dis	Korea	Bilateral facial nerve palsy; GBS
24	1	2007	Lee et al	J Korean Neurol Assoc	Korea	Guillain-Barre syndrome
25	1	2008	Lee et al	Diag Microbiol Infect Dis	Korea	Transversemyelitis
26	1	2008	Kim etal.	J Clin Neurol	Korea	Polyneuropathy; Cerebral Infarction
27	1	2009	Lee et al	Ir J Med Sci	Korea	Guillain-Barre syndrome
28	9	2009	Jim et al	Pediatr Neonatol	Taiwan	Meningitis
29	1	2009	Kang et al	BMC Infect Dis	Korea	Sensorineural hearing loss; otalgia
30	16	2009	Wu et al	Int J Gerontol	Taiwan	Meningitis ; meningoencephalitis
31	7	2010	Vivekanandan et al	J Assoc Physicians India	India	Meningitis
32	31	2010	Lee et al	BMC Infect Dis	Korea	Seizure;coma; confusion
33	23	2010	Kim et al	BMC Infect Dis	Korea	Meningoencephalitis
34	1	2010	Lee et al	J AAPOS	Korea	Isolated abducens palsy
35	1	2010	Oh et al	Infect Chemother	Korea	Bell's Palsy
36	4	2010	Mahajan et al	Emerg Infect Dis	India	Meningitis
37	6	2011	Thakur et al	J Laryngol Otol	India	Hearing loss
38	2	2011	Kang et al	Korean J Intern Med	Korea	Guillain-Barre syndrome
39	1	2011	Lin et al	Kaohsiung J Med Sci	Korea	Bilateral facial nerve palsy
40	1	2011	Kim et al	Diag Microbiol Infect Dis	Korea	Meningoencephalitis
41	1	2011	Yum et al	Clin Neurol Neurosurg	Korea	Meningoencephalitis
42	2	2012	Saifudheen et al	Ann Indian Acad Neurol	India	Meningoencephalitis
43	2	2012	Kumar et al	J Infect Pub Health	India	Meningitis
44	1	2012	Remalayam et al	J Postgrad Med	India	Subdural hematoma
45	9	2012	Khan et al	Int J Infect Dis	India	Coma, seizures

## Materials and Methods

The Pondicherry Institute of Medical Sciences is a 550- bedded teaching hospital serving the coastal town of Pondicherry and surrounding districts of Tamil Nadu with a population of nearly seven million. We did a retrospective analysis of all adult cases (≥16 years) of scrub typhus that were admitted in the hospital between February 2011 and January 2012. Computerized records of the Medical Records Department were searched using the terms, “scrub typhus”, “typhus”, “scrub typhus meningitis” and “rickettsial meningitis”. Confirmed cases of scrub typhus were selected based on a positive scrub IgM ELISA (Scrub Typhus Detect™ IgM ELISA, InBios India, detecting IgM antibodies to *Orientia tsutsugamushi* derived recombinant antigens), a positive Weil-Felix test (WFT), the presence of an eschar or a combination of the three in a patient with an acute febrile illness. Probable cases of scrub typhus without any of the above three were excluded from the study even if recovery following doxycycline was noted. Sixty nine adult cases were found- 65 of scrub typhus, three of Indian tick typhus and one of endemic typhus with none of the four having meningitis. There were no pediatric cases found during the study period. Sixty five cases were included in the study and divided into two groups based on the presence or absence of meningitis. The remaining four patients without scrub typhus (but positive titers of WFT OX:2, OX:19) were excluded from the study.

*Ethics statement:* Only demographic data of patients were stored in the hospital database to enable retrieval of files manually based on patient codes. Charts and discharge summaries were perused. All data were anonymously analysed without individual patient consent due to the retrospective nature of the study. The Institute Ethics Committee of the Pondicherry Institute of Medical Sciences waived the need for individual informed consent and approved the study.

Meningitis was defined by the presence of headache or nuchal rigidity with either altered sensorium or focal neurological deficits on history or examination and with CSF pleocytosis >5. Hypotension was defined by a systolic blood pressure of <90 mmHg. Tachycardia and tachypnea were identified if the pulse and respiratory rates were >100/min and >20/min respectively. Respiratory symptoms included sore throat, cough, expectoration, hemoptysis, wheeze and breathlessness. Abdominal symptoms consisted of nausea, vomiting, diarrhea, abdominal pain, constipation, jaundice and hematemesis. Urinary symptoms comprised of frequency, dysuria, and haematuria. Hepatic injury was defined by the presence of transaminases elevation (×3times). Renal injury and renal failure was defined by doubling and trebling of serum creatinine from baseline and was based on the RIFLE criteria. Anemia was defined as hemogloin <10 g/dL, leucocytosis was defined as total counts>10×10^9^/L and thrombocytopenia was defined as platelet counts<150×10^9^/L. Respiratory complication was present if the CXR had pleural effusion, interstitial pneumonitis or ARDS. Multi-organ dysfunction was present if more than two systems were involved (hepatic, renal, respiratory, thrombocytopenia and the nervous system). Demographic data, history, examination and investigations were noted and stored in an Excel spread sheet for analysis.

Statistical analysis was performed using IBM SPSS Statistics 19 for Windows. Numerical data was analysed by descriptive statistics. Independent–samples T test was performed for continuous variables and they were expressed as means± standard deviation. Chi square test (or Fischer's exact test) was performed for categorical data. Statistical significance was defined as p value<0.05.

## Results

Seventeen (26%) patients had clinical and laboratory evidence of meningitis and male: female ratio for this group was 10∶7(Table 2). The meningitis group also had a significantly higher percentage of patients with low grade fever and a lower percentage with respiratory symptoms. On examination the temperature, pulse and respiratory rates and incidence of crackles were significantly lower in patients with meningitis. Arthralgia, fatigue, edema, lymphadenopathy, pleural effusions, crackles and rhonchi were seen only in the control group but were not statistically significant. Mean duration of hospital stay was lower in patients without meningitis. Elevated urea, elevated total WBC counts in blood and a normal chest X-ray (CXR) were significantly associated with the presence of scrub meningitis (Table 3). A higher percentage of patients in the control group had elevated ESR, AST (×3times), bilirubin, GGT and WFT positivity (>1∶80) but without statistical significance. A positive WFT (≥1∶20) was seen in 33 patients (10/15 patients with meningitis) but only 7(7/15) patients had WFT titers >1∶80.

**Table 2:** Demography and clinical features of scrub typhus patients with and without meningitis at admission.

	Without meningitis n = 48(%)	With meningitis n = 17(%)	p value
Age[years]	41.27±14.64	41.82±17.67	0.900
Age >40years	22(45.8)	8(47.1)	0.931
Sex [M∶F]	23∶25	10∶7	0.440
Fever duration[days]	9.83±4.77	8.29±3.47	0.228
Fever >7days	29(64.4)	10(58.8)	0.908
Low grade fever	1(2.1)	4(23.5)	0.004*
Intermittent fever	40(83.3)	14(82.4)	0.926
Chills	46(95.8)	14(82.4)	0.073
Headache	26(54.2)	13(76.5)	0.107
Respiratory symptoms	23(47.9)	2(11.8)	0.009*
Abdominal symptoms	25(52.1)	12(70.6)	0.185
Urinary symptoms	6(12.5)	2(11.8)	0.936
Myalgia	14(29.2)	4(17.6)	0.353
Arthralgia	4(8.3)	0	0.219
Fatigue	4(8.3)	0	0.219
Temperature <100F	6(12.5)	6(35.3)	0.037*
Pulse [beats/min]	99.94±16.87	85.71±19.01	0.005*
Pulse>100 bpm	21(43.8)	3(17.6)	0.055
SBP[mmHg]	109.94±23.45	107.53±7.50	0.335
Hypotension <100 mmHg	7(14.6)	0	0.096
RR[breaths/min]	20.44±5.03	17.18±2.76	0.014*
RR>20/min	18(37.5)	1(5.9)	0.014*
Edema	4(8.3)	0	0.219
Lymphadenopathy	8(16.7)	0	0.072
Eschar	10(20.8)	3(17.6)	0.778
Diminished breath sounds	4(8.3)	0	0.219
Crackles	12(25)	0	0.022*
Rhonchi	5(10.4)	0	0.166
Hepatomegaly	20(41.7)	6(35.3)	0.645
Splenomegaly	11(22.9)	4(23.5)	0.238
Tender abdomen	4(8.3)	1(5.9)	0.745
Alcohol	8(16.7)	2(11.8)	0.630
Diabetes	6(12.5)	1(5.9)	0.102
Smoking	5(10.4)	2(11.8)	0.878
Hospital stay[days]	4.25±1.74	5.35±2.34	0.045*
Stay >1week	3(6.3)	2(11.8)	0.463

SBP-systolic blood pressure; RR-respiratory rate.

**Table 3:** Investigations of patients of scrub typhus with and without meningitis at admission.

Investigation	Without meningitis N = 48(%)	With meningitis N = 17(%)	p value
Creatinine µmol/L	89.46±47.90	83.70±31.40	0.646
Creatinine >88 µmol/L	16(33.3)	7(41.2)	0.561
Urea mmol/L	9.97±7.91	17.08±12.90	0.010*
Urea>7 mmol/L	25(52.1)	14(82.4)	0.029*
Renal injury	11(22.9)	5(29.4)	0.593
Renal failure	2(4.2)	0	0.393
Albumin<30 g/L	21(43.8)	9(52.9)	0.514
ALT µkat/L	2.00±1.39	2.21±2.64	0.690
ALT>2.1 µkat/L	16(33.3)	6(35.3)	0.883
AST µkat/L	1.96±1.55	1.67±2.79	0.603
AST>1.95 µkat/L	20(41.7)	4(23.5)	0.183
ALPµkat/L	2.31±1.69	2.34±1.24	0.953
ALP>1.65 µkat/L	22(45.8)	8(47.1)	0.931
GGT µkat/L	1.14±1.25	0.99±1.26	0.656
GGT>1 µkat/L	21(43.8)	6(35.3)	0.543
Bilirubin µmol/L	16.25±17.00	14.13±16.82	0.660
Bilirubin >22 µmol/L	11(24.4)	2(12.5)	0.672
Hemoglobin g%	11.60±2.33	11.89±2.54	0.664
Anemia <10 g%	13(27.1)	3(17.6)	0.438
Platelets ×10^9^/L	156.67±71.51	135.29±69.36	0.290
Platelets <150×10^9^/L	30(62.5)	11(64.7)	0.871
Total counts ×10^9^/L	8.32±2.91	13.02±7.88	0.028*
Total counts>10×10^9^/L	13(27.1)	8(47.1)	0.130
ESR mm/hour	30.46±23.73	38.88±28.45	0.237
ESR>30 mm	23(47.9)	4(23.5)	0.080
Albuminuria	9(18.8)	4(23.5)	0.672
Hematuria	4(8.3)	2(11.8)	0.674
Pyuria	9(18.8)	4(23.5)	0.672
MOD	24(50)	13(76.5)	0.058
WFT>1∶80 (n = 33)	18(81.8)	7(63.6)	0.251
Normal CXR	30(62.5)	15(88.2)	0.048*

ALT- alanine aminotransferase; AST-aspartate aminotransferase; ALP-alkaline phosphatase; GGT-gamma glutamyltransferase; MOD-multiorgan dysfunction; WFT-Weil-Felix test; CXR-chest x-ray; ESR-erythrocyte sedimentation rate.

December had the maximum admissions of scrub typhus, while the highest number of admissions with meningitis was in February (5/17). Unilateral and bilateral sixth cranial nerve palsies were the only cranial nerve deficits observed (Table 4). Altered sensorium was seen in five, while limb weakness was seen in three patients. Computed tomography (CT) brain of patients with altered sensorium was normal. Magnetic resonance imaging (MRI) could not be done due to lack of availability. Median cerebrospinal fluid (CSF) cell count, lymphocyte percentage, CSF protein, CSF glucose/blood glucose, CSF ADA was 54 cells/µL, 98%, 88 mg/dL, 0.622 and 3.5 U/mL respectively. All CSF samples were negative for gram stain, India ink, aerobic culture and acid fast bacilli. CSF pressure recordings were not performed.

**Table 4:** Clinical manifestations and CSF picture of patients scrub typhus with meningoencephalitis.

Patient	Age, gender	Neurological Deficits	Other Complications	Cells	Lym	Prot	Glu	CBS	ADA	WFT
1	29,F	-	Mastalgia	194	99	340	65	100	5.6	-
2	48,M	Irrelevant speech; abusive language; altered sensorium; power 4/5; normal CT brain	-	87	80	88	45	105	3.8	1∶320
3	51,M	-	-	54	68	167	80	106	3	1∶160
4	28,F	-	-	26	90	57	49	100		
5	30,F	U/L 6^th^ CNP; normal CT brain	-	158	100	200	70	100	1.6	1∶160
6	60,M	-	-	7	100	90	160	96	5.5	1∶160
7	27,F	-	-	18	90	35	74	108	1.6	1∶80
8	28,F		-	242	100	100	47	147	1.7	1∶20
9	30,F	-	-	15	90	71	57	98	1.8	1∶160
10	49,M		-	13	100	39	59	98	2.4	1∶80
11	58,F	-	ARF; LMZ pneumonitis	18	90	45	107	135	5	-
12	64,F	Altered sensorium; 3/5 power	Myocarditis; aspiration pneumonia	164	90	1150	34	255	3.7	1∶40
13	17,M	B/L 6^th^ CNP; B/L nystagmus; normal CT brain	-	387	100	63	84	115	3.5	-
14	17,M	Loss of consciousness	-	85	98	400	71	200	9.6	1∶40
15	38,M	-		10	100	32	70	132	1.8	-
16	65,M	Drowsiness; power 4/5	ARF	177	95	235	112	100	4.6	-
17	72,F	Altered sensorium	-	8	100	66	62	90	0.9	1∶40

CT-computed tomography; CNP-cranial nerve palsy; U/L-unilateral; B/L-bilateral; ARF-acute renal failure; LMZ-lower and middle zone; Lym-lymphocyte per cent; Prot-CSF protein; Glu-CSF glucose; CBS-corresponding blood sugar; ADA-adenosine deaminase; WFT- Weil Felix test.

Pleural effusion was observed in two females aged 23 and 40 years: thoracentesis revealed cell counts of 720 and 450 with lymphocyte percentages of 80% and 98%, pleural fluid protein 2 g and 2.2 g, fluid LDH/serum LDH values of 468/691[67%] and 527/1000[52.7%] and ADA values of 28 U and 76 U respectively. The first subject also had a posterior pericardial effusion. There was one woman (28 y) with 28 weeks' gestation, one case of acute pancreatitis following co-infection with dengue [18], one man with a papular rash, one woman (38 y) admitted under surgery for an acute abdomen and another woman (53 y) on antiretroviral therapy. HIV testing performed for lymphocytic meningitis was negative in all the 17 patients. All patients with meningitis had been treated with doxycycline (100 mg BD for 14 days) and a 51year old patient was given chloramphenicol in addition to doxycycline due to lack of defervescence.

## Discussion

In India, scrub typhus has been reported in at least 16 states (Jammu & Kashmir, Himachal Pradesh, Rajasthan, Haryana, Maharashtra, Karnataka, Andhra Pradesh, Kerala, Tamil Nadu, Pondicherry, West Bengal, Sikkim, Uttaranchal, Assam, Arunachal Pradesh, and Nagaland) [8],[19],[20]. Although most studies from Tamil Nadu are from one institute [21], documentation has been done in at least 15 districts [8],[21].

The differential diagnoses of scrub typhus in our setting include enteric fever, dengue, leptospirosis and malaria [22] all of which had been ruled out in our patients save one, who had a co-infection with dengue [18]. Clinical diagnosis is often made/confirmed by the presence of an eschar and clinical improvement following doxycycline therapy [22]. Eschars are seen in 7 to 80% of patients, with a higher percentage seen in children [14],[5]. Japan and Korea have a higher reportage of eschars when compared to Taiwan and Thailand [7]. Identification of eschars in the Indian population is difficult due to dark skin with incidence ranging from 4% to 46% [23],[24] and it was 20% in our study. Absence of an eschar was a risk factor for mortality [6]. Hepatomegaly and pneumonitis were the commonest manifestations in one study [5]. In our setting hepatomegaly and hepatic dysfunction were seen in 41.8% and 24.6% of subjects respectively while CXR abnormalities were observed in 20(30.8%) patients. Unusual manifestations include ARDS, myocarditis, pancreatitis, hemophagocytic syndrome, disseminated intravascular coagulation (DIC) and meningoencephalitis [25],[18]. Myocarditis, pleural effusion and gastrointestinal (GI) hemorrhage are more common with winter scrub typhus [12]. We had only one case each of myocarditis and hematemesis and two cases of pleural effusion. Hypoalbuminemia can be seen in one-fourth to three-fourths of patients of scrub typhus [26]. Perivasculitis, endothelial damage predisposes to capillary leak and hypoalbuminemia.

Scrub typhus involves both the central and peripheral nervous system. Tsutsugamushi is the rickettsia with the most meningeal involvement [27] but central nervous system(CNS) involvement is higher in epidemic typhus than that of scrub typhus [8]. CNS complications include infarction [28], cerebellitis [17], hemorrhages [29], encephalitis, demyelination [30], subdural hematoma [31], typhus nodules [32] and meningitis causing altered sensorium, agitation, motor weakness, seizures, neck stiffness, cranial nerve deficits(CND) [33]. Low platelets and DIC contribute towards haemorrhage [29]. Microglial nodules suggested cortical invasion [34]. The involved cranial nerves are the optic [17], abducens, facial and cochlear [35] nerves. Peripheral nervous system involvement reported are mononeuritis multiplex [36], brachial plexus neuropathy [37], polyneuropathy [28], myelitis [30], [38] and Guillain-Barre syndrome (GBS) [25].

Cranial nerve deficits are seen in ∼25% of patients with the sixth being most commonly involved [39]. Unilateral or bilateral abducens palsies occur with or without meningitis and facial palsies ensue singly or in association with GBS. Facial nerve palsy has also been described with *R. typhi* and *R. conorii* infections [25]. There is a ∼19% incidence of ear symptoms in scrub typhus that includes sensorineural hearing loss, otalgia and tinnitus [35]. Direct central nervous system invasion and involvement of the cochlear division or a secondary immune mediated effect in the vasovasorum of the cochlear nerve has been hypothesised [35]. Otalgia occurs in the first week, while sensorineural hearing loss and tinnitus occur during the second week [35]. Cochlear and retrocochlear damage has been proven histopathologically with demyelination and patchy neuritis having been noted in the 7^th^ and 8^th^ cranial nerves [9]. Lymphocytic infiltrate of the organ of Corti and the coclear nerve have been described [9]. Hearing loss has been reported in *R. conorii* [35].

The rickettsia directly invades the CSF and has been grown from CSF [25]. Nested PCR study revealed rickettsial DNA in CSF in patients with scrub meningitis [26]. A prospective study of Thai children revealed that scrub typhus was the second most common cause of aseptic meningitis next to Japanese encephalitis [40]. Meninoencephalitis is an autopsy finding in all fatal cases [34]. Involvement is generally due to leptomeningeal infiltration [32]. Histiocytes, lymphocytes and plasma cell infiltration of the meninges and perivascular spaces have been described. All patients with meningitis had received ceftriaxone 2 g Q12 hourly until serum IgM ELISA reports (or WFT) were available. None of our patients had had a repeat CSF study due to serological diagnosis and clinical recovery within 48 hours.

Most reports of meningitis are from Korea, India and Taiwan (Table 1). Meningeal signs were seen in 14% of patients with scrub typhus in a study conducted in Assam and Burma way back in 1946 [4]. In a study by Pai *et al.*, on 25 patients with CNS involvement, only half of them had CSF lymphocytosis and only a third had elevated protein [38]; *O. tsutsugamushi* DNA was isolated in six CSF samples. All our patients had CSF lymphocytic pleocytosis and a similar one-third had elevated protein (>60 mg). TBM remains the closest differential in our setting. India is one among the five countries that have the highest prevalence of TBM [39] with an estimated mortality of 1.5/100,000 population [41]. Staining for acid fast bacilli(AFB) in CSF has low sensitivity [42] and CSF culture for AFB takes up to 8 weeks and are positive only in 50–75% of cases [43],[44]. Hence other markers are necessary.

Adenosine deaminase(ADA) >10 increases the post-test probability of TBM [43]. Even though ADA is useful in a setting where TBM prevalence is low [43], we have used a similar cut-off in our patients with suspected TBM due to ready accessibility and low costs. CSF ADA levels for our patients were <10 U. Fever >7 days, CSF polys <50%, focal deficits, abnormal movements and optic atrophy had predicted the likelihood of TBM in a *Lancet* study [42]. Specificity and sensitivity were 98.3% and 54.5% if more than 3 variables were present and 98·4% and 43·5% if one or more predictors were present. Twelve of our patients had fever >7 days' duration, six had focal deficits and all of them had <50% CSF polys. In another model, disease duration>5 days, CSF lymphocytosis>70%, age>30 years and cells<1000 may predict TBM with sensitivity and specificity of 84% and 88% respectively [45]. Both models were based on studies in developing countries, but a single model may not predict TBM in all populations. Hence ADA levels may be helpful in differentiating scrub meningitis from tuberculous meningitis but more studies are necessary in that direction. Comparison with another group consisting of patients with tuberculous meningitis was not possible due to the retrospective study of scrub typhus patients alone. Prospective studies with cryptococcal antigen testing and PCR based kits like Xpert MTB/RIF (which were not performed in our patients due to unavailability) is needed in scrub meningitis to rule out other differential diagnoses. Scrub typhus meningitis can also be differentiated from TBM by the shorter period taken towards normalization [46]. Rifampicin is also used to treat severe scrub typhus and presence of lymphocytic CSF in a given patient, with improvement following antituberculous therapy (ATT) may mask the diagnosis of scrub typhus. Doxycycline remains the drug of choice, but azithromycin is used in pregnant patients and those with renal failure. In some instances, progressive neurological damage has occurred despite treatment [47], [48] with doxycycline either due to resistance, immune-mediated injury or due to drug interaction with oral antacids [49]. Doxycycline is bacteriostatic to *O. tsutsugamushi* and does not cross the blood brain barrier beyond 15–30%. Only one patient required chloramphenicol after 4 days of doxycycline therapy, but he had not been prescribed antacids. Recovery in meningoencephalitis is brisk with appropriate therapy. All neurological abnormalities in our study recovered within 2–5 days of doxycycline therapy. Patients were additionally administered dexamethasone and mannitol if they had altered sensorium or cranial nerve deficits. Delayed recovery over months in a case of isolated abducens palsy [48] and long term care in a patient with ADEM [30] have been described.

Specific tests for scrub typhus include indirect immunofluorescence test (IFA), immunoperoxidase test (IPT) and complement fixation test (CFT) [38]. IFA is the standard test for diagnosis, but lack of fluorescent microscopes makes it difficult for most hospitals [26]. IgM ELISA, based on the detection of 56 Da antigen [50] is a dot blot test which has high specificity (∼90%) and sensitivity (∼90%) when compared to IFA and IPT [26]. Scrub IgM was positive in all our patients. This test is expensive and ours is the only centre with this facility in this locality and surrounding districts. A two week course of doxycycline costs less than 50cents, while scrub IgM ELISA costs about 10–12 dollars and hence a cost-effectiveness trial in the future would be useful in our setting. On the other hand, a serological diagnosis may prevent prolonged treatment with ATT for suspected TBM. Also, a correct diagnosis could pre-empt a label of tuberculosis. A four-fold rise in titre is diagnostic, but it is retrospective and could not be performed due to costs. Thoracentesis in two patients revealed transudative fluid, but with a lymphocytic response and elevated ADA levels in one and borderline levels in the other. In such instances, differentiation from tuberculosis may be difficult. Leptospiral serology (IgM), malarial antigen testing, Widal, dengue IgM, IgG, Ns1 and blood culture was performed in all patients without an eschar. Since scrub IgM was performed in batches, and WFT was done in patients in whom a scrub IgM could not be expected within the next 1–3days. At a titre of ≥1∶80, sensitivity was 30% but specificity was nearly 100% for the WFT [50]. Also WFT is not useful until the second week of illness. Other advanced tests that have been used are the Sta56 gene amplification from eschars [51] and real time PCR studies based on the 56 kDa omp gene of the rickettsia, both of which would be difficult in the community setting. Pre antibiotic era mortality was >60% [52] and ∼30% in a 2006 series from Vellore [53] but with prompt diagnosis and therapy, the mortality now is very low. There were no deaths arising from scrub typhus during the duration of our study, partly contributed by earlier institution of doxycycline in patients with fever and multisystem involvement. Empirical treatment of fevers with chloramphenicol and tetracyclines and insect eradication measures could have led the disease to insignificance [50] after initial reports in India in the 1930s. But currently with increasing awareness, facilities for diagnosis, more literature is being published from India.

This study had some drawbacks. It was a retrospective study and data regarding occupation and place of origin were not available which would have given us the district-wise breakup of scrub typhus and the occupational risks associated with this illness. Also, time to defervescence has not been documented in most patients. Scrub IgM can be negative with some serotypes and hence cases of probable scrub typhus responsive to doxycycline that were excluded could have contributed numbers to the study. There was a referral bias due to availability of neurology/neurosurgery faculty, intensive care services and platelet transfusion accessibility that resulted in sicker patients and thrombocytopenic fevers being referred to our hospital especially by general practitioners and nursing homes. Differences in severity or differences in virulence occur among the various serotypes; confirming whether meningitis is caused only by some serotypes would have been ideal but could not be done due to dearth of facilities. Opening CSF pressure was not checked and whether they are elevated as in TBM is not known. Viral encephalitis, especially HSV related was not ruled out due to lack of diagnostic kits.

## Conclusion

Scrub typhus meningitis is a milder complication compared to respiratory or gastrointestinal problems even if it is associated with altered sensorium or cranial nerve deficits and generally resolves completely with doxycycline therapy. Due to the presence of lymphocytic pleocytosis with increased CSF protein, TBM is a close differential diagnosis. This may result in rifampicin based ATT masking the diagnosis of scrub typhus and sometimes results in patients continuing long term therapy for TBM. Since India is endemic for both TB and scrub typhus, awareness of simple-to-treat scrub typhus with access to specific tests like scrub IgM and CSF ADA may go a long way in avoiding unwarranted treatment in patients.
